# Three new *Diaporthe* species from Shaanxi Province, China

**DOI:** 10.3897/mycokeys.67.49483

**Published:** 2020-05-04

**Authors:** Qin Yang, Ning Jiang, Cheng-Ming Tian

**Affiliations:** 1 Key Laboratory for Non-Wood Forest Cultivation and Conservation of the Ministry of Education, Central South University of Forestry and Technology, Changsha 410004, China Beijing Forestry University Beijing China; 2 The Key Laboratory for Silviculture and Conservation of the Ministry of Education, Beijing Forestry University, Beijing 100083, China Central South University of Forestry and Technology Changsha China

**Keywords:** Diaporthaceae, Dieback, DNA phylogeny, Systematics, Taxonomy

## Abstract

*Diaporthe* species (Sordariomycetes, Diaporthales) are often reported as important plant pathogens, saprobes and endophytes on a wide range of plant hosts. In this study, *Diaporthe* specimens were collected from symptomatic twigs and branches at the Huoditang Forest Farm in Shaanxi Province, China. Identification was done using a combination of morphology and comparison of DNA sequence data of the nuclear ribosomal internal transcribed spacer (*ITS), calmodulin (cal*), histone H3 (*his3*), partial translation elongation factor-1α (*tef1*) and β-tubulin (*tub2*) gene regions. Three new *Diaporthe* species are proposed: *D. albosinensis*, *D. coryli* and *D. shaanxiensis*. All species are illustrated and their morphology and phylogenetic relationships with other *Diaporthe* species are discussed.

## Introduction

*Diaporthe* species (Sordariomycetes, Diaporthales) are associated with a wide range of plant hosts as pathogens, endophytes or saprobes of crops, ornamentals and forest trees (Murali et al. 2006, Rossman et al. 2007, Garcia-Reyne et al. 2011, Gomes et al. 2013, Udayanga et al. 2015, Dissanayake et al. 2017, Guarnaccia and Crous 2017, 2018, Wijayawardene et al. 2017, Yang et al. 2017a, b, 2018, Fan et al. 2018, Guarnaccia et al. 2018). The sexual morph of *Diaporthe* is characterised by immersed ascomata and an erumpent pseudostroma with elongated perithecial necks. Asci are unitunicate, clavate to cylindrical. Ascospores are fusoid, ellipsoid to cylindrical, hyaline, biseriate to uniseriate in the ascus, sometimes with appendages (Udayanga et al. 2011). The asexual morph is characterised by ostiolate conidiomata, with cylindrical phialides producing three types of hyaline, aseptate conidia (Udayanga et al. 2011, Gomes et al. 2013).

Species identification in *Diaporthe* has traditionally been based on host association, morphology and culture characteristics (Mostert et al. 2001, Santos and Phillips 2009, Udayanga et al. 2011), resulting in the description of over 200 species (Hyde et al. 2020). Multiple species of *Diaporthe* can colonise a single host and one species can be associated with different hosts (Santos and Phillips 2009, Diogo et al. 2010, Santos et al. 2011, Gomes et al. 2013). In addition, considerable within-species variability of phenotypic characters has been reported (Rehner and Uecker 1994, Mostert et al. 2001, Udayanga et al. 2011). Thus, a polyphasic taxonomic approach, based on multi-locus DNA data, morphology and ecology, has been increasingly employed for species boundaries in the genus *Diaporthe* (Gomes et al. 2013, Huang et al. 2013, 2015, Udayanga et al. 2014a, b, 2015, Fan et al. 2015, Du et al. 2016, Gao et al. 2016, 2017, Guarnaccia and Crous 2017, Guarnaccia et al. 2018, Long et al. 2019).

Huoditang is located in the middle part of the southern slope of the Qinling Mountains at 33°18'~33°28'N, 108°21'~108°29'E. It belongs to the transitional zone of the northern subtropical and warm temperate zone in China. The terrain is complex and the climate is changeable (Zhang and Cao 2007). The plant communities are complex and, as a result, species diversity of fungi in the forest area is high (Zhang and Cao 2007). During trips to collect forest pathogens causing dieback in Shaanxi Province, cankered branches with typical *Diaporthe* fruiting bodies were investigated and sampled. The aim of the present study was to identify these fungi, based on modern polyphasic taxonomic concepts.

## Materials and methods

### Isolates

Fresh specimens of *Diaporthe* were collected from symptomatic twigs or branches in Shaanxi Province (Table 1). Isolates were obtained by removing a mucoid spore mass from conidiomata and spreading the suspension on the surface of 1.8% potato dextrose agar (PDA) in a 9 cm diam. Petri dish. Petri dishes were incubated at 25 °C until spores germinated. Single germinating conidia were transferred on to new PDA plates, which were kept at 25 °C in the dark. Specimens are deposited in the Museum of the Beijing Forestry University (BJFC). Axenic cultures are maintained in the China Forestry Culture Collection Centre (CFCC).

### Morphological analysis

Morphological observations of the asexual morph in the natural environment were based on features of the fruiting bodies produced on infected plant tissues and micromorphology, supplemented by cultural characteristics. Conidiomata from tree barks were sectioned by hand, using a double-edged blade and structures were observed under a dissecting microscope. The gross morphology of fruiting bodies was recorded using a Leica stereomicroscope (M205 FA). Fungal structures were mounted in clear lactic acid and micromorphological characteristics were examined at 1000× magnification using a Leica compound microscope (DM 2500) with differential interference contrast (DIC) optics. Thirty measurements of each structure were determined for each collection. Colony characters and pigment production on PDA were noted after 10 d. Colony colours were described according to Rayner (1970).

### DNA extraction, PCR amplification and sequencing

Genomic DNA was extracted from colonies grown on cellophane-covered PDA, using the CTAB [cetyltrimethylammonium bromide] method (Doyle and Doyle 1990). PCR amplifications of phylogenetic markers were done using the same primer pairs and conditions as in Yang et al. (2018). PCR products were assayed via electrophoresis in 2% agarose gels. DNA sequencing was performed using an ABI PRISM 3730XL DNA Analyzer with a BigDye Terminater Kit v.3.1 (Invitrogen, USA) at the Shanghai Invitrogen Biological Technology Company Limited (Beijing, China).

### Phylogenetic analyses

The quality of our amplified nucleotide sequences was checked and combined by SeqMan v.7.1.0 and reference sequences were retrieved from the National Center for Biotechnology Information (NCBI), based on recent publications on the genus *Diaporthe* (Guarnaccia et al. 2018, Yang et al. 2018, Long et al. 2019). Sequences were aligned using MAFFT v. 7.310 (http://mafft.cbrc.jp/alignment/server/index.html) (Katoh and Standley 2016) and manually corrected using Bioedit 7.0.9.0 (Hall 1999). The best-fit nucleotide substitution models for each gene were selected using jModelTest v. 2.1.7 (Darriba et al. 2012) under the Akaike Information Criterion.

Phylogenetic analyses of the combined gene regions were performed using Maximum-Likelihood (ML) and Bayesian Inference (BI) methods. ML was conducted using PhyML v. 3.0 (Guindon et al. 2010), with 1000 bootstrap replicates. BI was performed using a Markov Chain Monte Carlo (MCMC) algorithm in MrBayes v. 3.0b4 (Ronquist and Huelsenbeck 2003). Two MCMC chains, started from random trees for 1,000,000 generations and trees, were sampled every 100th generation, resulting in a total of 10,000 trees. The first 25% of trees were discarded as burn-in of each analysis. Branches with significant Bayesian Posterior Probabilities (BPP) were estimated in the remaining 7500 trees. Phylogenetic trees were viewed with FigTree v.1.3.1 (Rambaut and Drummond 2010) and processed by Adobe Illustrator CS5. Alignment and trees were deposited in TreeBASE (submission ID: S25522). The nucleotide sequence data of the new taxa have been deposited in GenBank (Table 1).

**Table 1.** Isolates and GenBank accession numbers used in the phylogenetic analyses of *Diaporthe*.

Species	Isolate	Host	Location	GenBank accession numbers				
				ITS	*cal*	*his3*	*tef1*	*tub2*
*D. acericola*	MFLUCC 17-0956	*Acer negundo*	Italy	KY964224	KY964137	NA	KY964180	KY964074
*D. acerigena*	CFCC 52554	*Acer tataricum*	China	MH121489	MH121413	MH121449	MH121531	NA
***D. albosinensis***	**CFCC 53066**	***Betula albosinensis***	**China**	**MK432659**	**MK442979**	**MK443004**	**MK578133**	**MK578059**
	**CFCC 53067**	***Betula albosinensis***	**China**	**MK432660**	**MK442980**	**MK443005**	**MK578134**	**MK578060**
*D. alnea*	CBS 146.46	*Alnus* sp.	Netherlands	KC343008	KC343250	KC343492	KC343734	KC343976
*D. ambigua*	CBS 114015	*Pyrus communis*	South Africa	KC343010	KC343252	KC343494	KC343736	KC343978
*D. anacardii*	CBS 720.97	*Anacardium occidentale*	East Africa	KC343024	KC343266	KC343508	KC343750	KC343992
*D. angelicae*	CBS 111592	*Heracleum sphondylium*	Austria	KC343027	KC343269	KC343511	KC343753	KC343995
*D. apiculatum*	CGMCC 3.17533	*Camellia sinensis*	China	KP267896	NA	NA	KP267970	KP293476
*D. aquatica*	IFRDCC 3051	*Aquatic habitat*	China	JQ797437	NA	NA	NA	NA
*D. arctii*	CBS 139280	*Arctium lappa*	Austria	KJ590736	KJ612133	KJ659218	KJ590776	KJ610891
*D. aseana*	MFLUCC 12-0299a	Unknown dead leaf	Thailand	KT459414	KT459464	NA	KT459448	KT459432
*D. asheicola*	CBS 136967	*Vaccinium ashei*	Chile	KJ160562	KJ160542	NA	KJ160594	KJ160518
*D. baccae*	CBS 136972	*Vaccinium corymbosum*	Italy	KJ160565	NA	MF418264	KJ160597	NA
*D. beilharziae*	BRIP 54792	*Indigofera australis*	Australia	JX862529	NA	NA	JX862535	KF170921
*D. benedicti*	BPI 893190	*Salix* sp.	USA	KM669929	KM669862	NA	KM669785	NA
*D. betulae*	CFCC 50469	*Betula platyphylla*	China	KT732950	KT732997	KT732999	KT733016	KT733020
*D. betulina*	CFCC 52560	*Betula albo-sinensis*	China	MH121495	MH121419	MH121455	MH121537	MH121577
*D. bicincta*	CBS 121004	*Juglans* sp.	USA	KC343134	KC343376	KC343618	KC343860	KC344102
*D. caryae*	CFCC 52563	*Carya illinoensis*	China	MH121498	MH121422	MH121458	MH121540	MH121580
*D. cassines*	CPC 21916	*Cassine peragua*	South Africa	KF777155	NA	NA	KF777244	NA
*D. celeris*	CPC 28262	*Vitis vinifera*	Czech Republic	MG281017	MG281712	MG281363	MG281538	MG281190
*D. cercidis*	CFCC 52565	*Cercis chinensis*	China	MH121500	MH121424	MH121460	MH121542	MH121582
*D. chamaeropis*	CBS 454.81	*Chamaerops humilis*	Greece	KC343048	KC343290	KC343532	KC343774	KC344016
*D. charlesworthii*	BRIP 54884m	*Rapistrum rugostrum*	Australia	KJ197288	NA	NA	KJ197250	KJ197268
*D. chensiensis*	CFCC 52567	*Abies chensiensis*	China	MH121502	MH121426	MH121462	MH121544	MH121584
*D. cichorii*	MFLUCC 17-1023	*Cichorium intybus*	Italy	KY964220	KY964133	NA	KY964176	KY964104
*D. cinnamomi*	CFCC 52569	*Cinnamomum* sp.	China	MH121504	NA	MH121464	MH121546	MH121586
*D. citriasiana*	CGMCC 3.15224	*Citrus unshiu*	China	JQ954645	KC357491	KJ490515	JQ954663	KC357459
*D. citrichinensis*	CGMCC 3.15225	*Citrus* sp.	China	JQ954648	KC357494	NA	JQ954666	NA
*D. compactum*	CGMCC 3.17536	*Camellia sinensis*	China	KP267854	NA	KP293508	KP267928	KP293434
*D. conica*	CFCC 52571	*Alangium chinense*	China	MH121506	MH121428	MH121466	MH121548	MH121588
***D. coryli***	**CFCC 53083**	***Corylus mandshurica***	**China**	**MK432661**	**MK442981**	**MK443006**	**MK578135**	**MK578061**
	**CFCC 53084**	***Corylus mandshurica***	**China**	**MK432662**	**MK442982**	**MK443007**	**MK578136**	**MK578062**
*D. cucurbitae*	CBS 136.25	*Arctium* sp.	Unknown	KC343031	KC343273	KC343515	KC343757	KC343999
*D. cuppatea*	CBS 117499	*Aspalathus linearis*	South Africa	KC343057	KC343299	KC343541	KC343783	KC344025
*D. cynaroidis*	CBS 122676	*Protea cynaroides*	South Africa	KC343058	KC343300	KC343542	KC343784	KC344026
*D. cytosporella*	FAU461	*Citrus limon*	Italy	KC843307	KC843141	NA	KC843116	KC843221
*D. discoidispora*	ZJUD89	*Citrus unshiu*	China	KJ490624	NA	KJ490566	KJ490503	KJ490445
*D. dorycnii*	MFLUCC 17-1015	*Dorycnium hirsutum*	Italy	KY964215	NA	NA	KY964171	KY964099
*D. elaeagni-glabrae*	CGMCC 3.18287	*Elaeagnus glabra*	China	KX986779	KX999281	KX999251	KX999171	KX999212
*D. endophytica*	CBS 133811	*Schinus terebinthifolius*	Brazil	KC343065	KC343307	KC343549	KC343791	KC343065
*D. eres*	AR5193	*Ulmus* sp.	Germany	KJ210529	KJ434999	KJ420850	KJ210550	KJ420799
*D. eucalyptorum*	CBS 132525	*Eucalyptus* sp.	Australia	NR120157	NA	NA	NA	NA
*D. foeniculacea*	CBS 123208	*Foeniculum vulgare*	Portugal	KC343104	KC343346	KC343588	KC343830	KC344072
*D. fraxini-angustifoliae*	BRIP 54781	*Fraxinus angustifolia*	Australia	JX862528	NA	NA	JX862534	KF170920
*D. fraxinicola*	CFCC 52582	*Fraxinus chinensis*	China	MH121517	MH121435	NA	MH121559	NA
*D. fructicola*	MAFF 246408	*Passiflora edulis* × *P. edulis* f. *flavicarpa*	Japan	LC342734	LC342738	LC342737	LC342735	LC342736
*D. fusicola*	CGMCC 3.17087	*Lithocarpus glabra*	China	KF576281	KF576233	NA	KF576256	KF576305
*D. garethjonesii*	MFLUCC 12-0542a	*Unknown dead leaf*	Thailand	KT459423	KT459470	NA	KT459457	KT459441
*D. guangxiensis*	JZB320094	*Vitis vinifera*	China	MK335772	MK736727	NA	MK523566	MK500168
*D. helicis*	AR5211	*Hedera helix*	France	KJ210538	KJ435043	KJ420875	KJ210559	KJ420828
*D. heterophyllae*	CBS 143769	*Acacia heterohpylla*	France	MG600222	MG600218	MG600220	MG600224	MG600226
*D. hubeiensis*	JZB320123	*Vitis vinifera*	China	MK335809	MK500235	NA	MK523570	MK500148
*D. incompleta*	CGMCC 3.18288	*Camellia sinensis*	China	KX986794	KX999289	KX999265	KX999186	KX999226
*D. inconspicua*	CBS 133813	*Maytenus ilicifolia*	Brazil	KC343123	KC343365	KC343607	KC343849	KC344091
*D. infecunda*	CBS 133812	*Schinus terebinthifolius*	Brazil	KC343126	KC343368	KC343610	KC343852	KC344094
*D. juglandicola*	CFCC 51134	*Juglans mandshurica*	China	KU985101	KX024616	KX024622	KX024628	KX024634
*D. kadsurae*	CFCC 52586	*Kadsura longipedunculata*	China	MH121521	MH121439	MH121479	MH121563	MH121600
*D. litchicola*	BRIP 54900	*Litchi chinensis*	Australia	JX862533	NA	NA	JX862539	KF170925
*D. lusitanicae*	CBS 123212	*Foeniculum vulgare*	Portugal	KC343136	KC343378	KC343620	KC343862	KC344104
*D. masirevicii*	BRIP 57892a	*Helianthus annuus*	Australia	KJ197277	NA	NA	KJ197239	KJ197257
*D. middletonii*	BRIP 54884e	*Rapistrum rugostrum*	Australia	KJ197286	NA	NA	KJ197248	KJ197266
*D. millettiae*	GUCC9167	*Millettia reticulata*	China	MK398674	MK502086	NA	MK480609	MK502089
*D. miriciae*	BRIP 54736j	*Helianthus annuus*	Australia	KJ197282	NA	NA	KJ197244	KJ197262
*D. musigena*	CBS 129519	*Musa* sp.	Australia	KC343143	KC343385	KC343627	KC343869	KC344111
*D. neilliae*	CBS 144.27	*Spiraea* sp.	USA	KC343144	KC343386	KC343628	KC343870	KC344112
*D. neoarctii*	CBS 109490	*Ambrosia trifida*	USA	KC343145	KC343387	KC343629	KC343871	KC344113
*D. nothofagi*	BRIP 54801	*Nothofagus cunninghamii*	Australia	JX862530	NA	NA	JX862536	KF170922
*D. novem*	CBS 127270	*Glycine max*	Croatia	KC343155	KC343397	KC343640	KC343881	KC344123
*D. oraccinii*	CGMCC 3.17531	*Camellia sinensis*	China	KP267863	NA	KP293517	KP267937	KP293443
*D. ovalispora*	ICMP20659	*Citrus limon*	China	KJ490628	NA	KJ490570	KJ490507	KJ490449
*D. ovoicicola*	CGMCC 3.17093	*Citrus* sp.	China	KF576265	KF576223	NA	KF576240	KF576289
*D. osmanthi*	GUCC9165	*Osmanthus fragrans*	China	MK398675	MK502087	NA	MK480610	MK502090
*D. padina*	CFCC 52590	*Padus racemosa*	China	MH121525	MH121443	MH121483	MH121567	MH121604
*D. pandanicola*	MFLU 18-0006	*Pandanus* sp.	Thailand	MG646974	NA	NA	NA	MG646930
*D. pascoei*	BRIP 54847	*Persea americana*	Australia	JX862532	NA	NA	JX862538	KF170924
*D. passifloricola*	CBS 141329	*Passiflora foetida*	Malaysia	KX228292	NA	KX228367	NA	KX228387
*D. perseae*	CBS 151.73	*Persea gratissima*	Netherlands	KC343173	KC343415	KC343657	KC343899	KC344141
*D. pescicola*	MFLUCC 16-0105	*Prunus persica*	China	KU557555	KU557603	NA	KU557623	KU557579
*D. phaseolorum*	AR4203	*Phaseolus vulgaris*	USA	KJ590738	NA	KJ659220	NA	KP004507
*D. podocarpi-macrophylli*	CGMCC 3.18281	*Podocarpus macrophyllus*	China	KX986774	KX999278	KX999246	KX999167	KX999207
*D. pseudomangiferae*	CBS 101339	*Mangifera indica*	Dominican Republic	KC343181	KC343423	KC343665	KC343907	KC344149
*D. pseudophoenicicola*	CBS 462.69	*Phoenix dactylifera*	Spain	KC343184	KC343426	KC343668	KC343910	KC344152
*D. psoraleae-pinnatae*	CBS 136413	*Psoralea pinnata*	South Africa	KF777159	NA	NA	NA	KF777252
*D. pulla*	CBS 338.89	*Hedera helix*	Yugoslavia	KC343152	KC343394	KC343636	KC343878	KC344120
*D. racemosae*	CBS 143770	*Euclea racemosa*	South Africa	MG600223	MG600219	MG600221	MG600225	MG600227
*D. ravennica*	MFLUCC 15-0479	*Tamarix* sp.	Italy	KU900335	NA	NA	KX365197	KX432254
*D. rhusicola*	CBS 129528	*Rhus pendulina*	South Africa	JF951146	KC843124	NA	KC843100	KC843205
*D. rosae*	MFLU 17-1550	*Rosa* sp.	Thailand	MG828894	NA	NA	NA	MG843878
*D. rosicola*	MFLU 17-0646	*Rosa* sp.	UK	MG828895	NA	NA	MG829270	MG843877
*D. rudis*	AR3422	*Laburnum anagyroides*	Austria	KC843331	KC843146	NA	KC843090	KC843177
*D. sackstonii*	BRIP 54669b	*Helianthus annuus*	Australia	KJ197287	NA	NA	KJ197249	KJ197267
*D. salicicola*	BRIP 54825	*Salix purpurea*	Australia	JX862531	NA	NA	JX862537	JX862531
*D. sambucusii*	CFCC 51986	*Sambucus williamsii*	China	KY852495	KY852499	KY852503	KY852507	KY852511
*D. schini*	CBS 133181	*Schinus terebinthifolius*	Brazil	KC343191	KC343433	KC343675	KC343917	KC344159
*D. schoeni*	MFLU 15-1279	*Schoenus nigricans*	Italy	KY964226	KY964139	NA	KY964182	KY964109
*D. sennicola*	CFCC 51634	*Senna bicapsularis*	China	KY203722	KY228873	KY228879	KY228883	KY228889
*D. serafiniae*	BRIP 55665a	*Helianthus annuus*	Australia	KJ197274	NA	NA	KJ197236	KJ197254
***D. shaanxiensis***	**CFCC 53106**	**on branches of lian**a	**China**	**MK432654**	**MK442976**	**MK443001**	**MK578130**	**NA**
	**CFCC 53107**	**on branches of liana**	**China**	**MK432655**	**MK442977**	**MK443002**	**MK578131**	**NA**
*D. siamensis*	MFLUCC 10-573a	*Dasymaschalon* sp.	Thailand	JQ619879	NA	NA	JX275393	JX275429
*D. sojae*	FAU635	*Glycine max*	USA	KJ590719	KJ612116	KJ659208	KJ590762	KJ610875
*D. sterilis*	CBS 136969	*Vaccinium corymbosum*	Italy	KJ160579	KJ160548	MF418350	KJ160611	KJ160528
*D. stictica*	CBS 370.54	*Buxus sampervirens*	Italy	KC343212	KC343454	KC343696	KC343938	KC344180
*D. subclavata*	ICMP20663	*Citrus unshiu*	China	KJ490587	NA	KJ490529	KJ490466	KJ490408
*D. subcylindrospora*	MFLU 17-1195	*Salix* sp.	China	MG746629	NA	NA	MG746630	MG746631
*D. subellipicola*	MFLU 17-1197	on dead wood	China	MG746632	NA	NA	MG746633	MG746634
*D. subordinaria*	CBS 464.90	*Plantago lanceolata*	New Zealand	KC343214	KC343456	KC343698	KC343940	KC344182
*D. tectonendophytica*	MFLUCC 13-0471	*Tectona grandis*	China	KU712439	KU749354	NA	KU749367	KU749354
*D. tectonigena*	MFLUCC 12-0767	*Tectona grandis*	China	KU712429	KU749358	NA	KU749371	KU743976
*D. terebinthifolii*	CBS 133180	*Schinus terebinthifolius*	Brazil	KC343216	KC343458	KC343700	KC343942	KC344184
*D. ternstroemia*	CGMCC 3.15183	*Ternstroemia gymnanthera*	China	KC153098	NA	NA	KC153089	NA
*D. thunbergii*	MFLUCC 10-576a	*Thunbergia laurifolia*	Thailand	JQ619893	JX197440	NA	JX275409	JX275449
*D. tibetensis*	CFCC 51999	*Juglandis regia*	China	MF279843	MF279888	MF279828	MF279858	MF279873
*D. ueckerae*	FAU656	*Cucumis melo*	USA	KJ590726	KJ612122	KJ659215	KJ590747	KJ610881
*D. ukurunduensis*	CFCC 52592	*Acer ukurunduense*	China	MH121527	MH121445	MH121485	MH121569	NA
*D. unshiuensis*	CFCC 52594	*Carya illinoensis*	China	MH121529	MH121447	MH121487	MH121571	MH121606
*D. vaccinii*	CBS 160.32	*Oxycoccus macrocarpos*	USA	KC343228	KC343470	KC343712	KC343954	KC344196
*D. velutina*	CGMCC 3.18286	*Neolitsea* sp.	China	KX986790	NA	KX999261	KX999182	KX999223
*D. viniferae*	JZB320071	*Vitis vinifera*	China	MK341551	MK500107	NA	MK500119	MK500112
*D. xishuangbanica*	CGMCC 3.18282	*Camellia sinensis*	China	KX986783	NA	KX999255	KX999175	KX999216
*D. yunnanensis*	CGMCC 3.18289	*Coffea* sp.	China	KX986796	KX999290	KX999267	KX999188	KX999228
*Diaporthella corylina*	CBS 121124	*Corylus* sp.	China	KC343004	KC343246	KC343488	KC343730	KC343972

Newly sequenced material is indicated in bold type. NA, not applicable.

## Results

### Phylogenetic analyses

**Figure 1.**
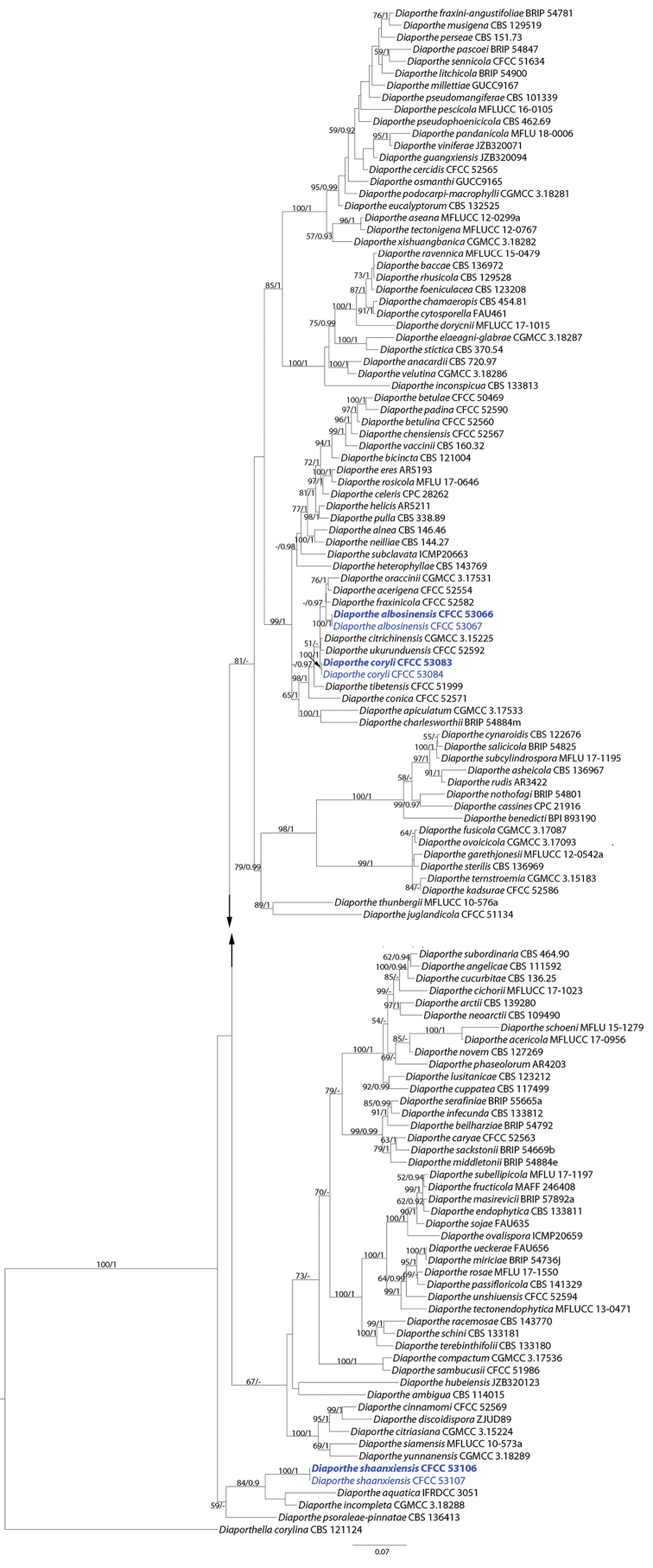
Phylogram of *Diaporthe* resulting from a maximum likelihood analysis based on combined ITS, *cal*, *his3*, *tef1* and *tub2*. Numbers above the branches indicate ML bootstraps (left, ML BS ≥ 50%) and Bayesian Posterior Probabilities (right, BPP ≥ 0.90). The tree is rooted with *Diaporthella corylina*. Isolates in current study are in blue. “-” indicates ML BS < 50% or BI PP < 0.90.

The five-gene sequence dataset (ITS, *cal*, *his3*, *tef1* and *tub2*) was analysed to infer the interspecific relationships within *Diaporthe*. The dataset consisted of 124 sequences including the outgroup, *Diaporthella corylina* (culture CBS 121124). A total of 2555 characters including gaps (505 for ITS, 513 for *cal*, 528 for *his3*, 475 for *tef1* and 522 for *tub2*) were included in the phylogenetic analysis. The best nucleotide substitution model for ITS, *his3* and *tub2* was TrN+I+G, while HKY+I+G was selected for both *cal* and *tef1*. The topologies resulting from ML and BI analyses of the concatenated dataset were congruent (Fig. 1). Isolates from Shaanxi Province formed three individual clades representing three undescribed species.

### Taxonomy

#### Diaporthe albosinensis

Taxon classificationFungiDiaporthalesDiaporthaceae

C.M. Tian & Q. Yang sp. nov.

27FA9968-ECF3-56FB-9BA6-7478C45CE54B

829518

Fig. 2

##### Diagnosis.

Distinguished from *D. fraxinicola* in having shorter conidiophores and longer beta conidia.

**Figure 2.**
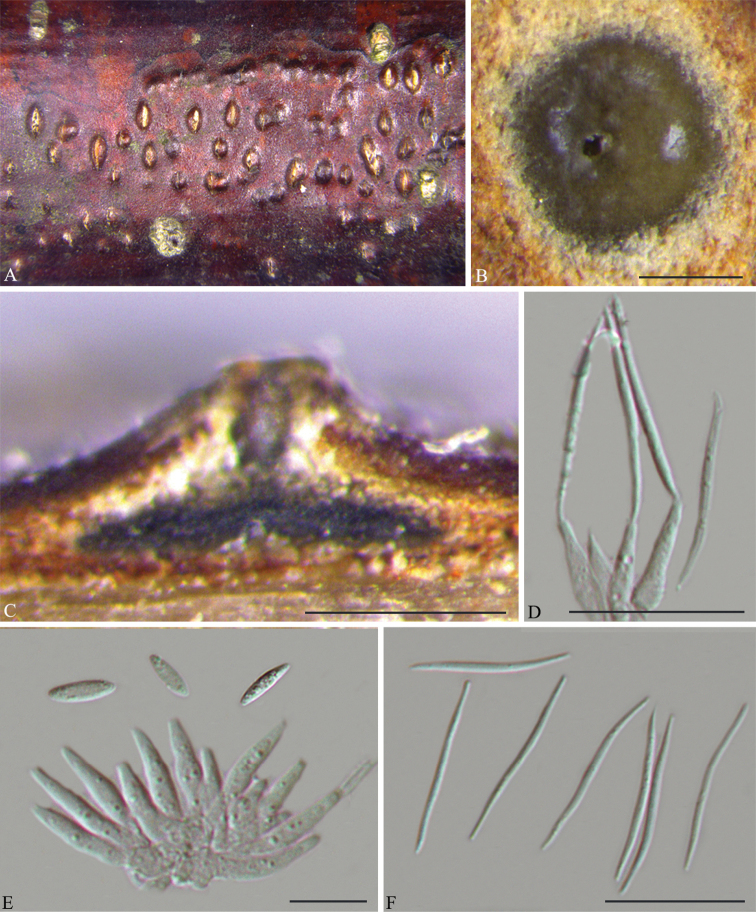
*Diaporthe albosinensis* on *Betula albosinensis* (BJFC-S1670). **A** Habit of conidiomata in wood **B** transverse section of conidiomata **C** longitudinal section through conidiomata **D** conidiogenous cells attached with beta conidia **E** conidiogenous cells attached with alpha conidia **F** beta conidia. Scale bars: 200 μm (**B–C**); 20 μm (**D, F**); 10 μm (**E**).

##### Etymology.

Named after the host plant, *Betula albosinensis*, from which the holotype was collected.

##### Description.

*Conidiomata* pycnidial, conical, immersed in bark, solitary to aggregated, erumpent through the bark surface, with a solitary undivided locule. *Ectostromatic disc* yellowish to brown, one ostiole per disc. *Ostiole* medium black, up to the level of disc. *Locule* undivided, (280–)290–375(–380) μm diam. *Conidiophores* (6–)8.5–13(–14.5) × (1.5–)2–2.5 μm, hyaline, cylindrical, smooth, phialidic, unbranched, straight or slightly curved. *Alpha conidia* hyaline, aseptate, fusiform, 0–1-guttulate, (7–)8–10(–11) × 2.5–3 μm. *Beta conidia* hyaline, aseptate, filiform, straight or slightly curved, eguttulate, base subtruncate, tapering towards one apex, (24–)25.5–30(–32) × 1–1.5 µm.

##### Culture characters.

Cultures incubated on PDA at 25 °C in the dark. Colony originally flat with white felted aerial mycelium, becoming light brown due to pigment formation, conidiomata irregularly distributed over agar surface, with yellowish conidial drops exuding from the ostioles.

##### Specimens examined.

China. Shaanxi Province: Ningshan County, Huoditang Forest Farm, 33°28'25"N, 108°29'39"E, on branches of *Betula albosinensis*, 10 July 2018, *N. Jiang* (holotype BJFC-S1670; ex-type living culture: CFCC 53066; living culture: CFCC 53067).

##### Notes.

Two isolates, representing *D. albosinensis*, are retrieved in a well-supported clade (ML BS/BPP=100/1) and appear most closely related to *D. fraxinicola* (Fig. 1). *Diaporthe albosinensis* can be distinguished from *D. fraxinicola*, based on *tef1* and *tub2* loci (3/335 in *tef1* and 19/429 in *tub2*). Morphologically, *D. albosinensis* differs from *D. fraxinicola* in having shorter conidiophores (8.5–13 vs. 10.5–17.5 μm) and longer beta conidia (25.5–30 vs. 19–29.5 μm) (Yang et al. 2018).

#### Diaporthe coryli

Taxon classificationFungiDiaporthalesDiaporthaceae

C.M. Tian & Q. Yang sp. nov.

0790CB77-EE63-5681-AD57-D83CB470F50D

829520

Fig. 3

##### Diagnosis.

Distinguished from *D. ukurunduensis* and *D. citrichinensis* in having larger alpha conidia.

**Figure 3.**
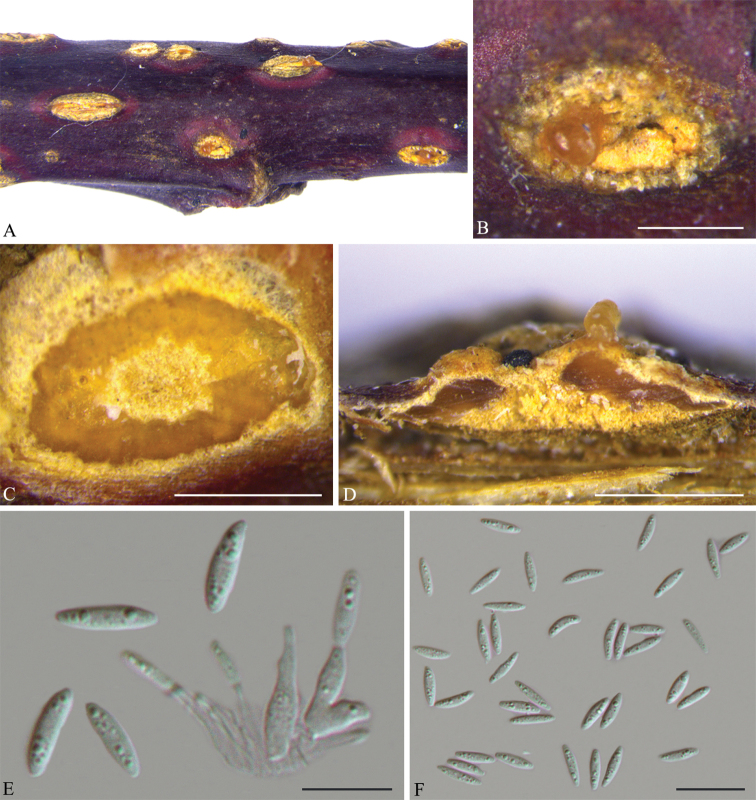
*Diaporthe coryli* on *Corylus mandshurica* (BJFC-S1671). **A, B** Habit of conidiomata in wood **C** transverse section of conidiomata **D** longitudinal section through conidiomata **E** conidiogenous cells attached with alpha conidia **F** alpha conidia. Scale bars: 500 μm (**B–D**); 10 μm (**E**); 20 μm (**F**).

##### Etymology.

Named after the genus of the host plant from which the holotype was collected, *Corylus*.

##### Description.

*Conidiomata* pycnidial, conical to spherical, immersed in the host bark, erumpent from surface of host branches, scattered, 950–1200 × 420–650 μm diam., covered by orange discharged conidial masses at maturity, usually conspicuous. *Ectostromatic disc* inconspicuous. *Central column* beneath the disc more or less conical, bright yellow. *Conidiophores* reduced to conidiogenous cells. *Conidiogenous cells* cylindrical, hyaline, smooth, unbranched, tapering towards the apex, (8.5–)10–12(–13) × (2–)2.5–3 μm. *Alpha conidia* hyaline, aseptate, fusiform, multiguttulate, rarely 2-guttulate, (10.5–)11.5–13(–13.5) × 3–3.5 μm. *Beta conidia* not observed.

##### Culture characters.

Cultures incubated on PDA at 25 °C in the dark. Colony flat, felty with thick texture at the marginal area, with thin texture in the centre, producing beige pigment after 7–10 d. Aerial mycelium white, dense, conidiomata distributed in the centre, with translucent conidial drops exuding from the ostioles.

##### Specimens examined.

CHINA. Shaanxi Province: Ningshan County, Huoditang Forest Farm, 33°28'26"N, 108°29'40"E, on branches of *Corylus mandshurica*, 10 July 2018, *N. Jiang* (holotype BJFC-S1671; ex-type living culture: CFCC 53083); 33°28'26"N, 108°29'38"E, on branches of *Corylus mandshurica*, 10 July 2018, *N. Jiang* (paratype BJFC-S1672; living culture: CFCC 53084).

##### Notes.

We generated sequences for two isolates of *D. coryli*, CFCC 53083 and CFCC 53084. This new species is phylogenetically most closely related to *D. ukurunduensis* and *D. citrichinensis* (Fig. 1). *Diaporthe coryli* can be distinguished from *D. ukurunduensis*, based on ITS, *his3* and *tef1* loci (8/467 in ITS, 1/460 in *his3* and 1/336 in *tef1*); and from *D. citrichinensis* based on *tef1* and *tub2* loci (4/335 in *tef1* and 25/428 in *tub2*). Morphologically, *D. coryli* can be distinguished from both *D. ukurunduensis* (11.5–13 × 3–3.5 vs. 5–6 × 2–3 μm) and *D. citrichinensis* (11.5–13 × 3–3.5 vs. 5.5–9 × 1.5–2.5 μm) in having larger alpha conidia (Huang et al. 2013, Gao et al. 2016).

#### Diaporthe shaanxiensis

Taxon classificationFungiDiaporthalesDiaporthaceae

C.M. Tian & Q. Yang sp. nov.

1B8F6C9B-59D3-5D30-B416-6175363932FD

829527

Fig. 4

##### Diagnosis.

Distinguished from *D. aquatica* and *D. incompleta* in having longer beta conidia.

**Figure 4.**
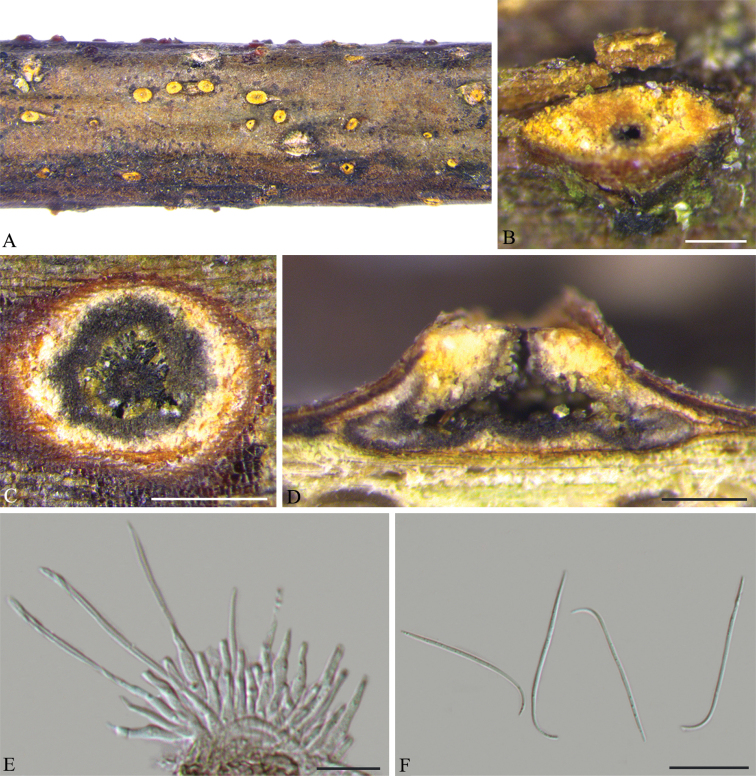
*Diaporthe shaanxiensis* on liana (BJFC-S1674). **A, B** Habit of conidiomata on twig **C** transverse section through conidiomata **D** longitudinal section through conidiomata **E** conidiogenous cells attached with beta conidia **F** beta conidia. Scale bars: 200 μm (**B–D**); 10 μm (**E, F**).

##### Etymology.

Named after Province Shaanxi, where the holotype was collected.

##### Description.

*Conidiomata* pycnidial, immersed in bark, scattered, erumpent through the bark surface, discoid, with a solitary undivided locule. *Ectostromatic disc* yellowish to pale brown, one ostiole per disc, usually conspicuous, (485–)500–687(–695) μm diam. *Locule* circular, undivided, (500–)526–765(–792) μm diam. *Conidiophores* reduced to conidiogenous cells. *Conidiogenous cells* hyaline, cylindrical, unbranched, slightly curved, tapering towards the apex, (12.5–)14.5–17(–18) × 1–1.5(–2) μm. *Alpha conidia* not observed. *Beta conidia* hyaline, aseptate, filiform, straight to curved, eguttulate, (35.5–)37–47.5(–50) × 1 µm.

##### Culture characters.

Cultures incubated on PDA at 25 °C in the dark. Colony originally flat with white fluffy aerial mycelium, becoming pale brown with pigment, with visible solitary conidiomata at maturity.

##### Specimens examined.

CHINA. Shaanxi Province: Ningshan County, Huoditang Forest Farm, 33°28'25"N, 108°29'39"E, on branch of liana, 10 July 2018, *N. Jiang* (holotype BJFC-S1674; ex-type living culture: CFCC 53106); 33°28'24"N, 108°29'38"E, on branch of liana, 10 July 2018, *N. Jiang* (Paratype BJFC-S1675; living culture: CFCC 53107).

##### Notes.

In the combined tree, *D. shaanxiensis* is a distinct clade with maximum support and it appears to be most closely related to *D. aquatica* and *D. incompleta* (Fig. 1). *Diaporthe shaanxiensis* can be distinguished from *D. aquatica* by a 17 nt difference in the ITS region. For *D. aquatica*, only ITS sequences are available in NCBI GenBank (Hu et al. 2012). The new species can be distinguished from *D. incompleta*, based on ITS, *cal*, *his3* and *tef1* (24/454 in ITS, 14/443 in *cal*, 66/468 in *his3* and 24/311 in *tef1*). Morphologically, *D. shaanxiensis* differs from both *D. aquatica* (37–47.5 vs. 9–12.5 µm) and *D. incompleta* (37–47.5 vs. 19–44 µm) in having longer beta conidia (Gao et al. 2016, 2017).

## Discussion

In this study, an investigation of forest pathogens from Huoditang in Shaanxi Province was carried out and Diaporthe canker was observed as a common disease. Identification of our collections was conducted, based on isolates from fruiting bodies using five combined loci (ITS, *cal*, *his3*, *tef1* and *tub2*), as well as morphological characters. Three new *Diaporthe* species were described. These are *D. albosinensis* sp. nov., *D. coryli* sp. nov. and *D. shaanxiensis* sp. nov.

*Diaporthe albosinensis* is associated with *Betula albosinensis*. Thus far, six *Diaporthe* species have been reported from *Betula*. These are *D. alleghaniensis*, *D. betulae*, *D. betulicola*, *D. betulina*, *D. eres* and *D. melanocarpa* (Kobayashi 1970, Gomes et al. 2013, Du et al. 2016, Yang et al. 2018). Morphologically, *D. albosinensis* differs from *D. betulae* (600–1250 μm), *D. betulicola* (700–1300 μm) and *D. betulina* (670–905 μm) in having smaller locules (Du et al. 2016, Yang et al. 2018); and from *D. alleghaniensis* (5–8 × 1.5–2 μm) and *D. eres* (6.5–8.5 × 3–4 μm) in having larger alpha conidia (Arnold 1967, Anagnostakis 2007, Gomes et al. 2013). In addition, our phylogenetic reconstruction of a five-locus dataset adds support for the new species, although no sequence data are currently available for *D. alleghaniensis*, *D. betulicola* and *D. melanocarpa* (Fig. 1). Interestingly, *D. melanocarpa* is found on different plant hosts; it was described from *Pyrus melanocarpa* in London and then recorded from *Amelanchier*, *Betula* and *Cornus* (Dearness 1926, Wehmeyer 1933, Kobayashi 1970). *Diaporthe coryli* is characterised by the ostiole with orange discharged conidial masses and a yellow central column (Fig. 3). *Diaporthe shaanxiensis* was found on branches of liana with an obvious ostiole per disc and characterised by hyaline, filiform beta conidia. Alpha conidia were found neither in the natural environment nor in culture for this species.

Species delimitation of *Diaporthe* has improved considerably by using a combination of morphological, cultural, phytopathological and molecular phylogenetic analyses (Udayanga et al. 2014a, b, 2015, Fan et al. 2015, Gao et al. 2017, Guarnaccia and Crous 2017, Hyde et al. 2017, 2020, Guarnaccia et al. 2018, Yang et al. 2018, Long et al. 2019). As a result, many Diaporthe canker diseases and new species have been discovered and reported from all over the world and also in China. The descriptions and molecular data of *Diaporthe* species represent an important resource for plant pathologists, plant quarantine officials and taxonomists.

## Supplementary Material

XML Treatment for Diaporthe albosinensis

XML Treatment for Diaporthe coryli

XML Treatment for Diaporthe shaanxiensis
